# Ruscogenin Ameliorates Experimental Nonalcoholic Steatohepatitis via Suppressing Lipogenesis and Inflammatory Pathway

**DOI:** 10.1155/2014/652680

**Published:** 2014-07-20

**Authors:** Hung-Jen Lu, Thing-Fong Tzeng, Shorong-Shii Liou, Chia Ju Chang, Cheng Yang, Ming-Chang Wu, I-Min Liu

**Affiliations:** ^1^Department of Food Science, College of Agriculture, National Pingtung University of Science and Technology, Neipu Township, Pingtung County, Taiwan; ^2^Department of Pharmacy and Graduate Institute of Pharmaceutical Technology, Tajen University, Yanpu Township, Pingtung County, Taiwan

## Abstract

The aim of the study was to investigate the protective effects of ruscogenin, a major steroid sapogenin in Ophiopogon japonicus, on experimental models of nonalcoholic steatohepatitis. HepG2 cells were exposed to 300 *μ*mol/l palmitic acid (PA) for 24 h with the preincubation of ruscogenin for another 24 h. Ruscogenin (10.0 *μ*mol/l) had inhibitory effects on PA-induced triglyceride accumulation and inflammatory markers in HepG2 cells. Male golden hamsters were randomly divided into five groups fed a normal diet, a high-fat diet (HFD), or a HFD supplemented with ruscogenin (0.3, 1.0, or 3.0 mg/kg/day) by gavage once daily for 8 weeks. Ruscogenin alleviated dyslipidemia, liver steatosis, and necroinflammation and reversed plasma markers of metabolic syndrome in HFD-fed hamsters. Hepatic mRNA levels involved in fatty acid oxidation were increased in ruscogenin-treated HFD-fed hamsters. Conversely, ruscogenin decreased expression of genes involved in hepatic lipogenesis. Gene expression of inflammatory cytokines, chemoattractive mediator, nuclear transcription factor-(NF-) *κ*B, and *α*-smooth muscle actin were increased in the HFD group, which were attenuated by ruscogenin. Ruscogenin may attenuate HFD-induced steatohepatitis through downregulation of NF-*κ*B-mediated inflammatory responses, reducing hepatic lipogenic gene expression, and upregulating proteins in *β*-oxidation pathway.

## 1. Introduction 

Nonalcoholic fatty liver disease (NAFLD) is a potentially severe condition that comprises a spectrum of pathologies characterized by vesicular fatty change in the liver in the absence of excessive alcohol consumption [1]. NAFLD is strongly associated with obesity, insulin resistance, and type 2 diabetes and is now well recognized as being part of the metabolic syndrome [2]. The metabolic pathways leading to the development of hepatic steatosis are multiple, including enhanced nonesterified fatty acid release from adipose tissue (lipolysis), increased* de novo* fatty acids (lipogenesis), and decreased *β*-oxidation. To date, caloric restriction and aerobic exercise are effective treatments for NAFLD, but they are difficult to achieve for most NAFLD patients. Other than lifestyle and diet modifications, there is no universally proven treatment [3]. Therefore, the development of additional therapies for controlling lipid levels is warranted to attenuate hepatic steatosis.

NAFLD is classified into simple steatosis and nonalcoholic steatohepatitis (NASH). In NASH, not only steatosis but also intralobular inflammation and hepatocellular ballooning are present, often accompanied by progressive fibrosis [1]. A variety of liver cells such as hepatocytes, hepatic macrophages, and hepatic stellate cells (HSCs) are involved in the pathogenesis of NASH [4]. Among the inflammatory mediators, chemokines play pivotal roles in the recruitment of a variety of cells including immune cells to the sites of inflammation through interaction with chemokine receptors [5]. The interaction of cytokines/growth factors with their receptors initiates different signaling pathways, leading to the activation of multiple transcriptional factors such as nuclear transcription factor- (NF-) *κ*B, which also has a role in liver fibrogenesis [6]. Therapies that limit hepatic injury and the related occurrence of inflammation and fibrosis are particularly appealing for this condition.

**Figure 1:**
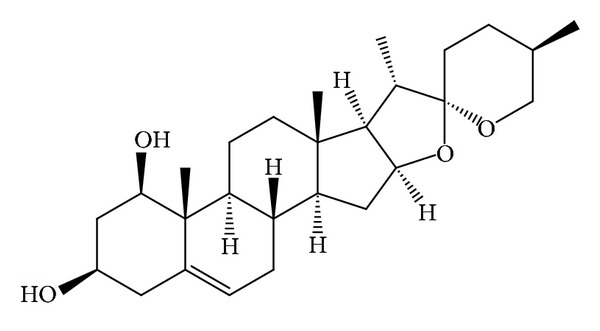
Structure of ruscogenin.

Ruscogenin ((1*β*, 3*β*, 25R)-Spirost-5-ene-1,3-diol; Figure 1) is a major effective steroidal sapogen in the traditional Chinese herb Radix* Ophiopogon japonicus*, that has been used to treat acute and chronic inflammatory and cardiovascular diseases [7, 8]. Ruscogenin has been found to exert significant anti-inflammatory activities in many aspects such as antielastase activity, inhibiting leukocyte adhesion and migration, and antithrombotic activity [9–11]. Previous research has proved that the possible anti-inflammatory mechanism of ruscogenin was linked with the suppression of intercellular adhesion molecule-1 expression in endothelial cells mainly through the inhibition of the NF-*κ*B signaling pathway [12]. It was also found that ruscogenin significantly attenuated lipopolysaccharide- (LPS-) induced acute lung injury model in mice, which possibly linked with inhibition of NF-*κ*B activation [13]. Ruscogenin has also been documented to reduce cerebral ischemic injury via NF-*κ*B mediated inflammatory pathway in the mouse model of experimental stroke. [14]. Downregulation of NF-*κ*B-mediated inflammatory responses induced by ruscogenin may indicate its potential protection in NASH; however, there is no report about it until now. Therefore, the aim of the present study was to investigate the protective effects of ruscogenin supplementation on liver steatosis and injury in hamsters fed a high-fat diet (HFD). These effects of ruscogenin were further characterized in HepG2 hepatocytes.

## 2. Materials and Methods

### 2.1. Cell Cultures

Human hepatoma HepG2 cells, obtained from Bioresource Collection and Research Center (BCRC 60025) of the Food Industry Research and Development Institute (Hsinchu, Taiwan), were cultured in minimum essential medium containing 10% (vol/vol) fetal bovine serum, 2 mmol/L l-glutamine, 1 mmol/L sodium pyruvate, 100 U/mL penicillin, 100 *μ*g/mL streptomycin, and 1 mmol/L sodium pyruvate at 37°C in a humidified atmosphere containing 5% CO_2_. The cells were grown to 70% confluence and incubated in serum free medium overnight before treatments. After the starvation for 24 h, cells were exposed to 300 *μ*mol/L palmitic acid (PA; Sigma-Aldrich Co., St. Louis, MO; Cat. no. P0500) for 24 h with or without the preincubation of 0.1, 1.0, or 10.0 *μ*mol/L ruscogenin (≥98%; Chengdu Biopurify Phytochemicals Ltd., Chengdu, Sichuan, China; Cat. no. 472-11-7) for another 24 h. Cell viability after PA treatments was monitored by trypan blue exclusion. No change in viability was observed with the concentrations used in this study. Subconfluent monolayers of HepG2 cells were stained with Oil Red O (Sigma-Aldrich Co.) to determine fat accumulation.

### 2.2. Observation of PA-Induced Lipids Accumulation in HepG2 Cells

The PA-induced lipid accumulation in HepG2 cells was evaluated by Oil Red O staining and the measurement of triglyceride (TG) content. Briefly, samples were fixed with 4% paraformaldehyde and then stained with Oil Red O for 15 min. Then, the samples were counterstained with hematoxylin for 5 min. Results were examined by light microscopy. Intracellular TG content was evaluated after lysis of the cells with cell lysis buffer (1% Triton X-100, 150 mmol/L NaCl, 10 mmol/L Tris, pH 7.4, 1 mmol/L EDTA, 1 mmol/L EGTA, 0.2 mmol/L phenylmethylsulfonyl fluoride, 0.2 mmol/L sodium orthovanadate, and 0.5% NP-40) (Promega, Madison, WI, USA). The concentration of TG was determined by the Triglyceride Colorimetric Assay Kit (Cat. no. 10010303; Cayman Chemical Company, Ann Arbor, Michigan, USA) according to the protocol provided by manufacturer and normalized by protein content.

### 2.3. Measurement of PA-Induced Inflammatory Cytokines in HepG2 Cells

The cell culture media were centrifuged at 10,000 g for 10 min at 4°C and the supernatants were stored at −20°C before analysis. Secretory levels of inflammatory cytokines, including monocyte chemoattractant protein- (MCP-) 1 (Cat. no. ab100721), tumor necrosis factor- (TNF-) *α* (Cat. no. ab46070), interleukin- (IL-) 1*β* (Cat. no. ab100768), and IL-6 (Cat. no. ab100772), in cell-free culture supernatants were determined by commercial enzyme-linked immunosorbent assay (ELISA) kits purchased from Abcam Inc. (Cambridge, MA, USA). The color generated was determined by measuring the OD value at 450 nm of spectrophotometric microtiter plate reader (Molecular Devices Corp., Sunnyvale, CA, USA). A standard curve was run on each assay plate using recombinant proteins in serial dilutions.

### 2.4. Animal Models

Male golden Syrian hamsters, 8-week old and weighing 90 ± 10 g, were obtained from the National Laboratory Animal Center (Taipei, Taiwan). They were maintained in a temperature-controlled room (25 ± 1°C) on a 12 h : 12 h light-dark cycle (lights on at 06 : 00 h) in our animal center. Food and water were provided* ad libitum*. A regular chow diet (RCD; 10% kcal fat, no. D12450B; Research Diets, New Brunswick, NJ) was used as the maintenance and control diet. A purified HFD with 45% kcal fat obtained primarily from lard (no. D12451, Research Diets) was used to induce a rapid increase in body weight and obesity [15]. All animal procedures were performed according to the Guide for the Care and Use of Laboratory Animals of the National Institutes of Health, as well as the guidelines of the Animal Welfare Act. These studies were conducted with the approval of the Institutional Animal Care and Use Committee (IACUC) at Tajen University (approval number, IACUC 101-29; approval date, December 22, 2012).

### 2.5. Treatment Protocols in Animals

After being fed a HFD for two weeks, hamsters were dosed by oral gavage once per day for eight weeks with ruscogenin doses of 0.3, 1.0, or 3.0 mg/kg in a volume of 1.5 mL/kg distilled water. The dosage regimen was selected based on a previous report demonstrating that ruscogenin at the indicated dosage regimen was potentially effective in inhibiting LPS-induced inflammation in mice [13]. Another group of HFD- and RCD-fed hamsters was treated similarly, but the same volume of vehicle (distilled water) was used to prepare the tested compound solution during the same treatment period. The water consumption, food intake, and body weight were measured once daily at the same time (09 : 00) on each day throughout the experiment. Food cups containing fresh food were weighed at the beginning and end of each 24 h period. Food intake was calculated by determining the difference in food cup weights, adjusting for any spillage that occurred. Water intake was calculated by measuring the difference in water bottle weights at the beginning and end of the daily change of water.

Eight weeks after treatment with ruscogenin, animals were weighed and anesthetized with ketamine after fasting for 12 hours. Blood samples were taken from the inferior vena cava for analysis. After blood collection, liver, epididymal adipose, and perirenal adipose tissues were removed, rinsed with a physiological saline solution, and immediately stored at −80°C in liquid nitrogen until assayed. The coefficient of hepatic weight was also calculated as liver weight (g) divided by body weight (100 g). Other hepatic tissues were fixed in 10% neutralized formalin for histology.

### 2.6. Determination of Metabolic Parameters and Insulin Sensitivity

Blood samples were centrifuged at 2,000 ×g for 10 minutes at 4°C. The plasma was then removed and placed into aliquots for the respective analyses. Kits for determining plasma glucose (Cat. no. 10009582) concentration were purchased from Cayman Chemical Company (Ann Arbor, MI). Commercial ELISA kits were used to quantify plasma insulin concentration (LINCO Research, Inc., St. Charles, MO; Cat. no. EZRMI-13 K). Whole body insulin sensitivity was estimated using the homeostasis model assessment of insulin resistance (HOMA-IR) with the following formula: [fasting plasma glucose (mmol) × fasting plasma insulin (mU/mL)]/22.5 [16]. Diagnostic kits for determination of plasma levels of total cholesterol (TC; Cat. no. 10007640) and TG (Cat. no. 10010303) were purchased from Cayman Chemical Company. The diagnostic kit for determination of plasma levels of high density lipoprotein cholesterol (HDL-C) was purchased from Bio-Quant Diagnostics (Cat. no. BQ 019CR; San Diego, CA, USA). Low density lipoprotein cholesterol (LDL-C) concentrations in plasma were determined by commercial ELISA kit (antibodies-online Inc., Atlanta, GA, USA; Cat. no. ABIN416222). Plasma free fatty acid (FFA) levels were determined using an FFA quantification kit obtained from Abcam plc (Cat. no. ab65341; Cambridge, MA, USA). All experimental assays were carried out according to the manufacturer's instruction. All samples were analyzed in triplicate.

### 2.7. Measurement of Hepatic Lipids

Sections of fresh liver samples were collected for determining the lipid content. Liver (1.25 g) was homogenized with chloroform/methanol (1 : 2, 3.75 mL). Chloroform (1.25 mL) and distilled water (1.25 mL) were then added to the homogenate and mixed well. After centrifugation (1500 ×g for 10 min), the lower clear organic phase of the solution was transferred into a new glass tube and then lyophilized. The lyophilized powder was dissolved in chloroform/methanol (1 : 2) and stored at −20°C for less than 3 days [17]. Hepatic TC and TG levels in lipid extracts were analyzed using the same diagnostic kits that were used for plasma analysis.

### 2.8. Histological Analysis of Liver

At sacrifice, livers were perfused with phosphate buffered saline (PBS) solution via the portal vein. After removal of the liver, a section of approximately 4 mm^2^ was fixed in PAF and embedded in paraffin. Paraffin-embedded sections (5 *μ*m) were stained with hematoxylin and eosin (H&E) to evaluate the degree of hepatic steatosis. Liver tissues were scored for hepatic steatosis (0, none; 1, 1–25%; 2, 26–50%; 3, 51–75%; and 4, 76–100% hepatocytes affected) and necroinflammation (0, no inflammation; 1, mild lobular/portal inflammation; 2, moderate lobular/portal inflammation; and 3, severe lobular/portal inflammation) [18]. Other frozen liver sections were stained with macrophage-specific monoclonal antibody F4/80 (1 : 500, Cat. no. sc-377009; Santa Cruz Biotechnology, Santa Cruz, CA, USA) followed by detection with biotinylated secondary antibody and streptavidin-horseradish peroxidase to evaluate the degree of macrophage infiltration and fibrosis. All slides were scanned at a total magnification of 200x using Image Pro Plus 7.0 software (Media Cybernetics) under a light microscope (Olympus BX51 microscope; Tokyo, Japan).

### 2.9. Analysis of mRNA Expression of Hepatic Genes

For analysis of gene expression, total RNA was extracted from 100 mg frozen liver samples using Trizol reagent (Invitrogen; Boston, MA, USA). RNA was quantified by A260 and its integrity verified by agarose gel electrophoresis using ethidium bromide for visualization. For the reverse transcriptase reaction, 1 *μ*g of total RNA per sample and 8.5 *μ*g/*μ*L random hexamer primers were heated at 65°C for 5 min and then quenched on ice. This mixture was combined with 500 *μ*mol/L of each of dATP, dTTP, dCTP, and dGTP, 10 mmol/L DTT, 20 mmol/L Tris-HCl (pH 8.4), 50 mmol/L KCl, 5 mmol/L MgCl_2_, 40 units of RNaseOUT recombinant ribonuclease inhibitor (Invitrogen), and 100 units SuperScript III reverse transcriptase (Invitrogen). Samples were subjected to DNase (Promega; Madison, WI, USA) treatment at 37°C for 20 min in a GeneAmp 9700 Thermal Cycler (Applied Biosystems; Foster City, California, USA) and then held at 4°C. After aliquots were taken for immediate use in PCR, the remainder of the cDNA was stored at −20°C. mRNA expression was measured by quantitative real-time reverse transcription polymerase chain reaction (RT-PCR) in a fluorescent temperature Lightcycler 480 (Roche Diagnostics; Mannheim, Germany). The following primer sequences were used: 5′-TCTCTTCCTCCACCACTATGCA-3′(forward) and 5′-GGCTGAGACAGCACGTGGAT-3′ (reverse) for MCP-1; 5′-ATGGATCTCAAAGACAACCA-3′ (forward) and 5′-TCCTGGTATGAAATGGCAAA-3′(reverse) for TNF-*α*; 5′-GGTCAAAGGTTTGGAAGCAG-3′ (forward) and 5′-TGTGAAATGCCACCTTTTGA-3′(reverse) for IL-1*β*; 5′-AAAAGTCCTGATCCAGTTC-3′ (forward) and 5′-GAGATGAGTTGTCATGTCC-3′(reverse) for IL-6; 5′-TGCTGTCCCTCTATGCCTCT-3′ (forward) and 5′-GAAGGAATAGCCACGTCAG-3′(reverse) for *α*-smooth muscle actin (*α*-SMA); 5′-GAAGAAAATGGTGGAGTCTG-3′ (forward) and 5′- GGTTCACTAGTTTCCAAGTC-3′(reverse) for nuclear transcription factor-*κ*B (NF-*κ*B)/p65; 5′-CGTCCTGGCCTTCTAAACGTAG-3′ (forward) and 5′-CCTGTAGATCTCCTGCAGTAGCG-3′ (reverse) for peroxisome proliferator-activated receptor (PPAR) *α*; 5′-CAGACCTGACACAACACGG-3′ (forward) and 5′-CTTGAAAAATTGCTTGCGTC-3′ (reverse) for PPAR*γ* coactivator-1*α* (PGC-1*α*); 5′-ATGGCAGAGGCTCACCAAGCTGTG-3′ (forward) and 5′-CCTCTGTGGTACACAACAATGTGC-3′ (reverse) for carnitine palmitoyltransferase (CPT)-1; 5′-ATGGTTGGACTGAAGCCTTCAG-3′ (forward) and 5′-TCA AAACGGTGATTCCCGTAAC-3′ (reverse) for uncoupling protein (UCP)2; 5′-GAGGAGGAGGGATTCTGGTC-3′ (forward) and 5′-CACGTCTCCAACCTTCCATT-3′ (reverse) for UCP3; 5′-TCAACAACCAAGACAGTGACTTCCCTG GCC-3′ (forward) and 5′-GTTCTCCTGCTTGAGCTTCTGGTTGCTGTG-3′ (reverse) for sterol regulatory element-binding protein (SREBP)-1c; 5′-TCGTGGGCTACAGCATGGT-3′ (forward) and 5′-GCCCTCTGAAGTCGAAGAAGAA-3′ (reverse) for fatty acid synthase (FAS); 5′-CTGTAGAAACCCGGACAGTAGAAC-3′ (forward) and 5′-GGTCAGCATACATCTCCATGTG-3′ (reverse) for acetyl-CoA carboxylase (ACC); 5′-TGTGATGGTGGGAATGGGTCAG-3′ (forward) and 5′-TTTGATGTCACGCACGATTTCC-3′ (reverse) for *β*-actin. Primers were designed using Primer Express Software version 2.0 System (Applied Biosystems; Foster City, CA, USA). The PCR reaction was performed using the following cycling protocol: 95°C for 5 min, followed by 45 cycles of 95°C for 5 s, 58°C for 15 s, and 72°C for 20 s. Dissociation curves were run after amplification to identify the specific PCR products. The mRNA expression levels were normalized to *β*-actin mRNA levels and calculated according to the delta-delta Ct method [19].

### 2.10. Statistical Analysis

Data are expressed as the mean ± standard error mean (mean ± SEM). Statistical analysis was performed with one-way analysis of variance (ANOVA). Dunnett range post hoc comparisons were used to determine the source of significant differences, where appropriate. For the histological study, a nonparametric Kruskal-Wallis test was performed and Mann-Whitney's *U* test was used to compare data within the groups. The SigmaPlot (Version 11.0) program was used for statistical analysis. A *P* value < 0.05 was considered statistically significant.

## 3. Results

### 3.1. Effects of Ruscogenin on PA-Induced Lipids Accumulation and Inflammatory Cytokines in HepG2 Cells

**Figure 2:**
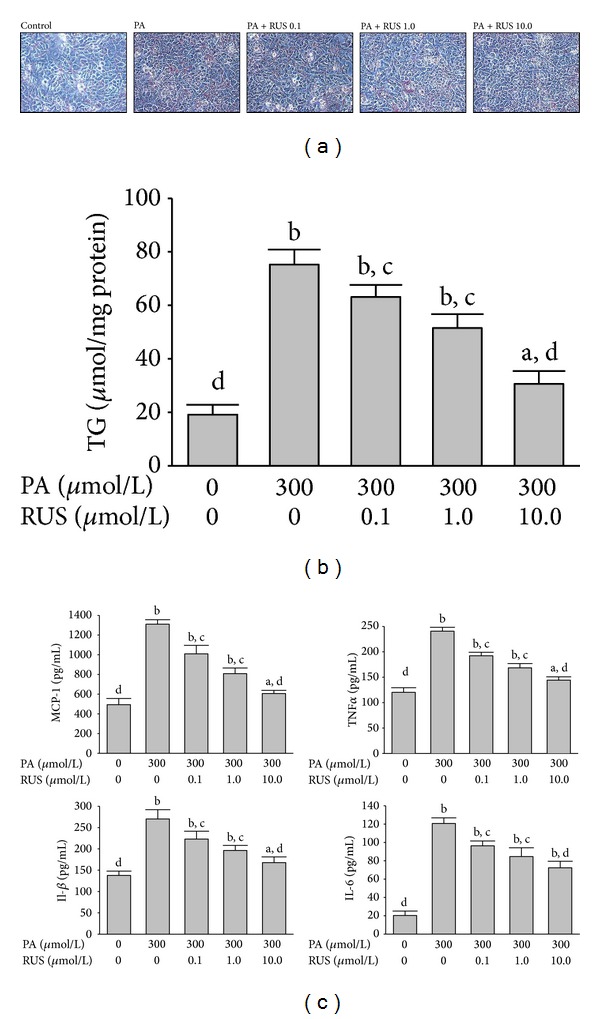
Effects of ruscogenin (RUS) on PA-induced lipids accumulation and inflammatory cytokines overproduction in HepG2 cells. Cells were exposed to PA (300 *μ*mol/L) for 24 h with or without the preincubation of 0.1 (RUS 0.1), 1.0 (RUS 1.0), or 10.0 *μ*mol/L ruscogenin (RUS 10.0). (a) Representative Oil Red O staining of cells with different treatments is shown. Cells were examined by light microscopy at a magnification of 400x. (b) Intracellular TG content was measured by an ELISA assay. TG concentration was normalized by protein content. (c) Inflammatory cytokines in cell-free culture supernatants were determined by ELISA kits. The results are presented as the mean ± SEM of four experiments. ^a^ *P* < 0.05 and ^b^ *P* < 0.01 compared to the control values of untreated cells (control), respectively. ^c^ *P* < 0.05 and ^d^ *P* < 0.01 compared to the values of PA-treated cells (PA), respectively.

The lipid accumulation was measured by Oil Red O staining. As shown in Figure 2(a), HepG2 cells treated for 24 h with PA exhibited significant lipid droplet accumulation compared with untreated cells. Preincubation with ruscogenin significantly prevented PA-induced lipid deposition and the most effective inhibition of lipid accumulation occurred at a dose of 10 *μ*mol/L (Figure 2(a)). Consistently, treatment with PA resulted in an obvious increase in TG content compared with untreated cells, which was attenuated significantly by pretreatment with ruscogenin at a concentration of 10 *μ*mol/L (Figure 2(b)). Ruscogenin alone or vehicle did not affect basal levels of lipid deposition (data not shown).

Figure 2(c) showed that PA exposure significantly increased the production of MCP-1, TNF-*α*, IL-1*β*, and IL-6 compared with the untreated cells. Pretreatment with 10 *μ*mol/L ruscogenin for 24 h significantly alleviated PA-induced overproduction of all these inflammatory cytokines.

### 3.2. Effects of Ruscogenin on the Body Weight, the Liver and Visceral Fat Weight, and Feeding Behaviors of Hamsters

The body weight, visceral fat, and relative liver weights in HFD-fed hamsters were significantly higher than those in the RCD-fed group (Table 1). High doses of ruscogenin (3.0 mg/kg/day) significantly suppressed body weight gain. The relative weights of liver, epididymal adipose tissue, and perirenal adipose tissue were significantly lower in ruscogenin (3.0 mg/kg/day) treated groups than those in the HFD group (Table 1). No significant differences in daily food or water intake were observed between the groups over the experimental period (Table 1).

### 3.3. Effects of Ruscogenin on Insulin Sensitivity and Plasma Lipids Levels of Hamsters

HFD-fed hamsters were insulin resistant as reflected by hyperinsulinemia as well as significantly increased HOMA-IR values (Table 2). Treatment of HFD-fed hamsters with ruscogenin (3.0 mg/kg/day) had a beneficial effect on insulin resistance, as evidenced by a reduction in fasting plasma insulin levels, and improved levels of HOMA-IR (Table 2).

The HFD caused elevated concentrations of plasma TC, TG, and LDL. Oral administration of ruscogenin at a dose of 0.3, 1.0, or 3.0 mg/kg/day significantly reduced plasma TC levels (23.7%, 31.1%, and 39.7% reduction, resp.); the reduction of plasma TG activity induced by ruscogenin at 3.0 mg/kg/day was nearly 30.1% (Table 2). Ruscogenin at the oral dose of 0.3, 1.0, or 3.0 mg/kg/day significantly reduced total plasma LDL levels (32.9%, 42.6%, and 54.3% reduction, resp.) compared to that of vehicle-treated HFD-fed hamsters (Table 2).

The plasma concentration of HDL-C in HFD-fed hamsters was reduced to 60.4% of the level observed in the RCD-fed group (Table 2). After 8 weeks of ruscogenin (3.0 mg/kg/day) treatment, plasma HDL-C concentrations in HFD-fed hamsters increased to 90.5% of the level in the RCD-fed group (Table 2).

Plasma FFA levels in vehicle-treated HFD-fed hamsters were about 2.0-fold of that observed in the RCD-fed group (Table 2). The plasma FFA levels decreased by 38.1% in HFD-fed hamsters treated with ruscogenin (3.0 mg/kg/day) compared to their vehicle-treated counterparts (Table 2).

### 3.4. Effects of Ruscogenin on Hepatic Steatosis

The hepatic TC level was significantly higher in HFD-fed hamsters than in hamsters from the RCD-fed group, which was reduced by 28.5% in HFD-fed hamsters treated with ruscogenin (3.0 mg/kg/day; Table 2). Similarly, ruscogenin treatment (3.0 mg/kg/day) also produced a significant reduction in hepatic TG concentration to 63.2% of that in vehicle-treated HFD-fed hamsters (Table 2).

**Figure 3:**
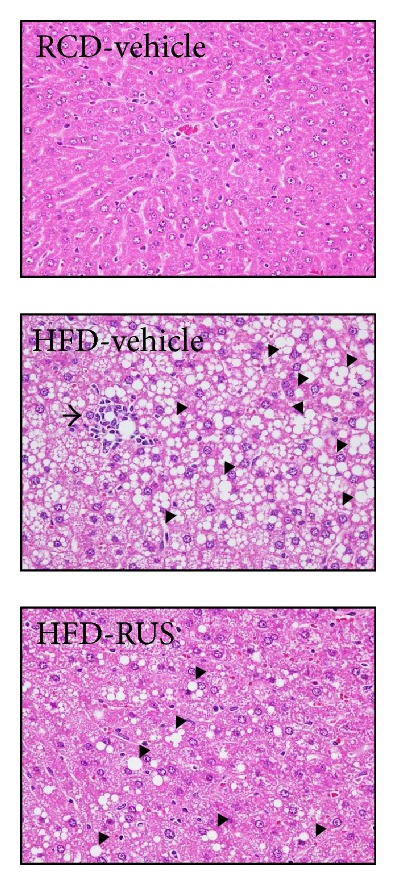
Representative images of H&E and stained livers from RCD- or HFD-fed hamsters receiving 8 weeks of treatments. Photomicrographs (original magnification, 400x) are of tissues isolated from vehicle-treated RCD-fed hamsters (RCD-vehicle), vehicle-treated HFD-fed hamsters (HFD-vehicle), or ruscogenin (3.0 mg/kg/day) treated HFD-fed hamsters (HFD-ruscogenin). Arrows and arrow head indicate fat droplets and necroinflammatory foci, respectively. The severity of hepatic steatosis and necroinflammation were scored in Table 3.

Representative histological photomicrographs of liver specimens are shown in Figure 3. Hamsters fed a RCD had normal liver histological findings; however, numerous macrovascular fat droplets and mild necroinflammatory foci were present in livers of those fed a HFD. Treatment of HFD-fed hamsters with ruscogenin (3.0 mg/kg/day) reduced fat liver depots and less macrovesicular steatosis as revealed in vehicle-treated counterparts (Figure 3). Ruscogenin treatment (3.0 mg/kg/day) clearly reduced hepatic necroinflammation (Figure 3). Histological grading of liver sections confirmed that ruscogenin treatment significantly ameliorated both hepatic steatosis and necroinflammation (Table 3).

**Figure 4:**
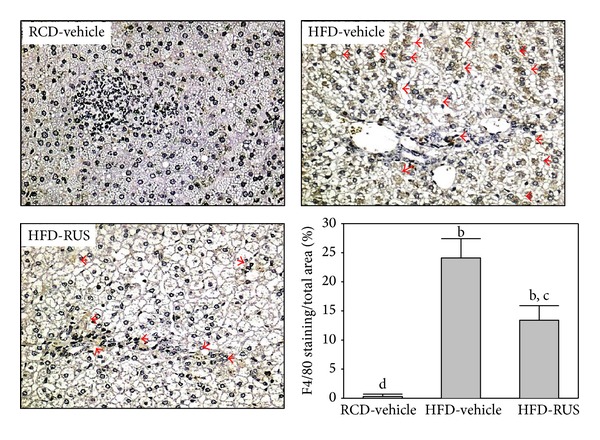
Representative images of F4/80 staining in livers from RCD- or HFD-fed hamsters receiving 8 weeks of treatments. Photomicrographs are of tissues isolated from vehicle-treated RCD-fed hamsters (RCD-vehicle), vehicle-treated HFD-fed hamsters (HFD-vehicle), or ruscogenin (3.0 mg/kg/day) treated HFD-fed hamsters (HFD-ruscogenin). Arrows indicate inflammatory foci. Quantification of hepatic macrophage accumulation is presented as the percentage of the brown stained area relative to the whole area of the photomicrograph (original magnification, 400x). Values (mean ± SEM) were obtained from 5 animals in each group. ^b^ *P* < 0.01 compared to vehicle-treated RCD-fed hamsters. ^c^ *P* < 0.05 and ^d^ *P* < 0.01 compared to the values of vehicle-treated HFD-fed hamsters in each group, respectively.

Livers from RCD-fed hamsters did not show any significant macrophage (F4/80-positive cells) infiltration (Figure 4). In contrast, HFD-fed hamsters demonstrated prominent macrophage infiltration of the liver (Figure 4). Treatment of HFD-fed hamsters with ruscogenin (3.0 mg/kg/day) for 8 weeks showed a marked reduction in macrophage influx by 29.3%, when compared with their vehicle-treated counterparts (Figure 4).

### 3.5. Effects of Ruscogenin on Inflammatory Cytokines in Hamsters

**Figure 5:**
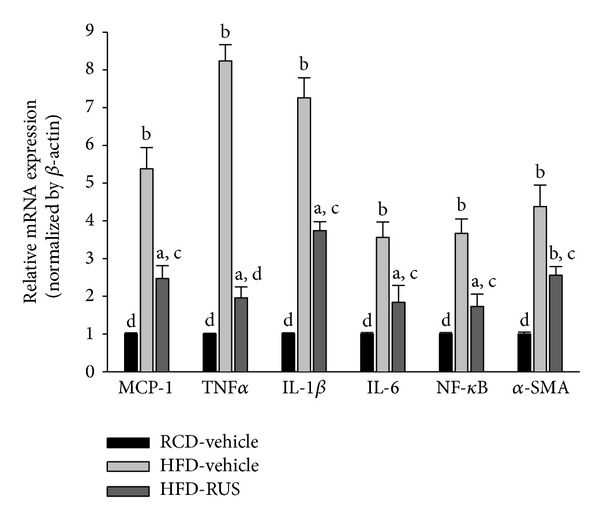
Real-time PCR analysis of mRNA expression of NF-*κ*B-dependent proinflammatory markers in the livers of HFD-fed hamsters receiving 8 weeks of treatment with ruscogenin (3.0 mg/kg/day). The mRNA expressions of the inflammatory cytokines and NF-*κ*B were normalized to an internal control (*β*-actin). Animals not receiving any treatment were given the same volume of vehicle used to dissolve ruscogenin. Similar results were obtained with an additional 4 replications. Data were expressed as the mean with SEM (*n* = 5 per group) in each column. ^a^ *P* < 0.01 and ^b^ *P* < 0.01 compared to vehicle-treated RCD-fed hamsters. ^c^ *P* < 0.05 and ^d^ *P* < 0.01 compared to the values of vehicle-treated HFD-fed hamsters in each group, respectively.

All inflammatory mediators were expressed at very low levels in the livers of RCD-fed hamsters (Figure 5). In HFD-fed hamsters, hepatic mRNA levels of MCP-1, TNF-*α*, IL-1*β*, IL-6, and NF-*κ*B were significantly increased compared to RCD-fed group (Figure 5). These increases were approached to control values in hamsters treated with ruscogenin. In addition, the hepatic fibrosis index of *α*-SMA in HFD-fed hamsters was significantly increased to 4.3-fold of that observed in the RCD-fed group and decreased (41.5% decreases) by ruscogenin treatment (Figure 5).

### 3.6. Effects of Ruscogenin on Hepatic mRNA Expression of *β*-Oxidation-Related Genes and Lipogenic Genes

**Figure 6:**
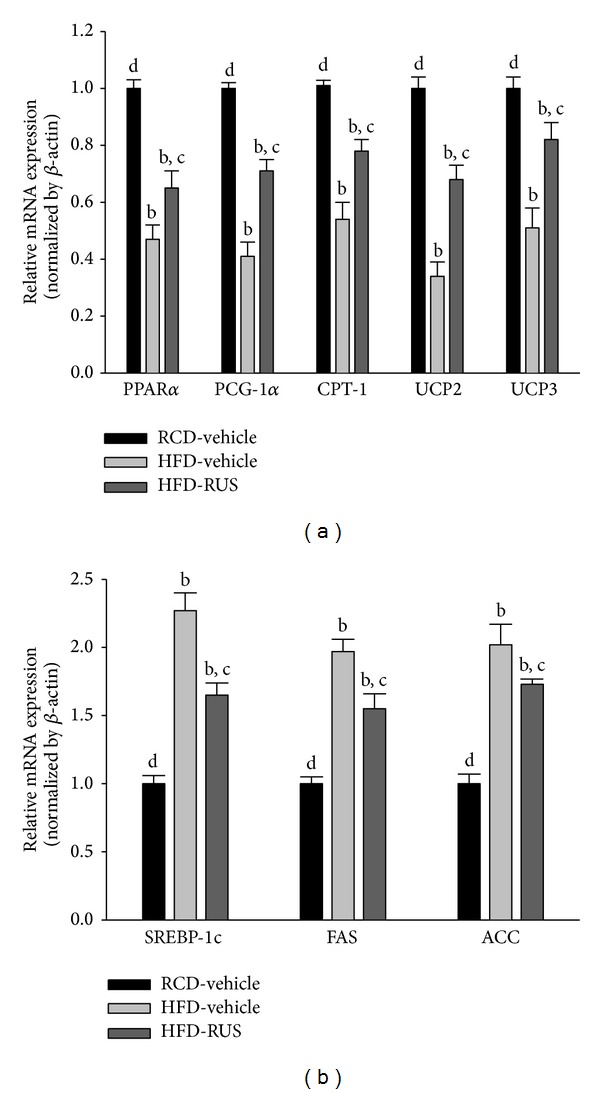
The hepatic mRNA levels of *β*-oxidation-related genes (a) and lipogenic genes (b) in HFD-fed hamsters receiving 8 weeks of treatment with ruscogenin (3.0 mg/kg/day; HFD-RUS). The mRNA expressions of the *β*-oxidation-related genes and lipogenic genes were measured by RT-PCR and normalized to an internal control (*β*-actin). Animals not receiving any treatment were given the same volume of vehicle used to dissolve ruscogenin. Similar results were obtained with an additional 4 replications. Data were expressed as the mean with SEM (*n* = 5 per group) in each column. ^b^ *P* < 0.01 compared to vehicle-treated RCD-fed hamsters (RCD-vehicle). ^c^ *P* < 0.05 and ^d^ *P* < 0.01 compared to the values of vehicle-treated HFD-fed hamsters (HFD-vehicle) in each group, respectively.

The mRNA levels of PPAR*α* in livers of HFD-fed hamsters were decreased to 38.2% of those of the RCD-fed group (Figure 6(a)). Administration of ruscogenin to HFD-fed hamsters for 8 weeks significantly upregulated hepatic PPAR*α* mRNA levels to 1.7-fold that of vehicle-treated counterparts (Figure 6(a)). Hepatic mRNA levels of PGC-1*α*, CPT-1, UCP2, and UCP3 in HFD-fed hamsters were clearly lower than those of the RCD-fed group and were upregulated by ruscogenin treatment (173.2, 144.3, 163.2, and 198.3% increases, resp.) (Figure 6(a)).

HFD feeding markedly increased the hepatic mRNA levels of SREBP-1c in hamsters to 2.3-fold that of the RCD-fed group (Figure 6(b)). Ruscogenin suppressed the HFD-induced increase in hepatic mRNA levels of SREBP-1c by 27.3% relative to vehicle-treated counterparts (Figure 6(b)). HFD caused a 1.9-fold induction of hepatic FAS mRNA and a 2.0-fold induction of hepatic ACC mRNA over those of the RCD-fed group (Figure 6(b)). The HFD-induced mRNA levels of FAS and ACC in liver were significantly reversed after ruscogenin treatment (decreased by 21.3 and 28.5%, resp.) compared to those of vehicle-treated counterparts (Figure 6(b)).

## 4. Discussion

As inflammation plays a pivotal role in NAFLD, an important pharmacological objective in treating this disorder is the direct targeting of inflammatory activation [5]. Because PA is known to induce a hyperlipidemic condition viainflammatory liver injury [20, 21], it could be thought that PA treatment sufficiently caused hepatic steatosis and inflammation. Our data demonstrated that exposure of HepG2 cells to PA results in lipid accumulation and the overproduction of inflammatory cytokines such as MCP-1, TNF-*α*, IL-1*β*, and IL-6. This is in agreement with a previous study that the plasma concentrations of FFAs were increased in patients with NAFLD and correlated with the development of more severe liver disease [22]. Our study clearly demonstrated that preincubation with ruscogenin significantly attenuates the lipid accumulation and inflammatory cytokines overproduction in HepG2 cells exposed to excess PA. These data indicated that ruscogenin might be effective for preventing and reversing lipid accumulation and the inflammatory response which may accelerate the lipid metabolism disorder and/or more severe liver injuries.

The HFD-induced animal model of NAFLD has been widely used to study its pathogenesis and to evaluate new treatments [23]. We then investigated whether ruscogenin could improve fatty liver changes by decreasing inflammatory cytokines in the HFD-induced NAFLD hamsters. With ruscogenin treatment of HFD-fed hamsters, we observed that the increased plasma levels of TG, TC, LDL-C, and FFA were significantly suppressed, whereas the decreased plasma HDL-C levels were clearly elevated. In addition, the relative liver weight of ruscogenin-treated hamsters was significantly lower than that of HFD-fed hamsters. Morphologically, the livers of HFD-fed hamsters showed large, abundant lipid droplets and clear derangement compared to those of RCD-fed hamsters. However, the livers of HFD-fed hamsters receiving ruscogenin had fewer lipid droplets and more normal liver morphology, suggesting that ruscogenin had the beneficial effects of preventing lipid accumulation and reversing disrupted liver structure.

Hepatic macrophages are the key cells inducing liver inflammation, and infiltration of hepatic macrophages was increased in hamsters on HFD. Inflammation and subsequent chemoattraction of cell migration to the liver are critical for the development and progression of NAFLD [24]. Administration of ruscogenin during NAFLD development significantly attenuated reduced hepatic macrophage infiltration as well as inflammatory mediators in HFD-fed hamsters. Molecules that initiate hepatic fibrosis may also be activated by excessive FFAs through lipid peroxidation or cytokine production [5]. In addition, activated hepatic Kupffer cells, the direct source of proinflammatory cytokine production, may in turn activate HSC to synthesize collagen, initiating the process of liver remodeling in the form of fibrosis and cirrhosis [5, 6]. Expression of *α*-SMA has been considered one of the dominant features of HSC activation and has become an important evaluation index for hepatic fibrosis [25]. We observed that the expression of *α*-SMA induced by HFD was significantly reduced by ruscogenin, suggesting that the inhibition of inflammatory factor expression also effectively suppressed HSC activation, blocking the occurrence of hepatic fibrosis at the source.

Since NF-*κ*B is the master regulator of molecules that take part in cellular proliferation, inflammation, and apoptosis, blockade of NF-*κ*B pathway has shown therapeutic efficacy [6]. The previous studies have demonstrated that ruscogenin had significant anti-inflammatory and antithrombotic activities, which might be related to the inhibition of ICAM-1 and NF-*κ*B pathways [12]. The inhibition of NF-*κ*B by ruscogenin might be a critical step for the prevention of cascading inflammatory response in the liver. In the present study, ruscogenin suppressed the mRNA expression of NF-*κ*B in liver of HFD-fed hamsters, reflecting the effective blockage of HFD-induced NF-*κ*B activation. These results suggest that ruscogenin inhibits the activation of NF-*κ*B, leading to downregulation of proinflammatory mediators and amelioration of fibrogenesis, and therefore shows a promising effective in preventing NAFLD.

Importantly, insulin resistance is further associated with the development of steatosis and liver fibrosis [26]. Besides the direct effects of inflammatory cytokines on lipogenesis and fibrogenesis, MCP-1, TNF-*α*, and IL-1*β*, IL-6 can induce insulin resistance [24, 26]. Therefore, patients with type 2 diabetes are at a higher risk of NAFLD and other inflammatory processes. We observed that the effects of ruscogenin improved HOMA-IR in HFD-fed hamsters. These results further suggest that ruscogenin not only suppresses the recruitment of macrophages but also inhibits the release of inflammatory cytokines from hepatic macrophages, preventing hepatic steatosis, fibrosis, and insulin resistance. According to our results, ruscogenin could positively influence fatty liver changes in type 2 diabetes and in insulin resistant state.

Hepatic lipid metabolism is mainly regulated by lipid regulatory proteins, such as *β*-oxidation-related and lipogenic proteins. Expression of fatty acid *β*-oxidation proteins is the indicator of higher *β*-oxidation rates and attenuates hepatic lipid accumulation [27, 28]. In contrast, lowered expression of lipogenic proteins and SREBP-1c, an important lipid synthetic transcription factor, mediates hypolipidemic effects of lipid lowering agents [29–31]. To explore the possible mechanisms whereby ruscogenin decreases hepatic lipid accumulation, we investigated the expression levels of several genes related to lipid metabolism. Hepatic mRNA levels involved in fatty acid oxidation (PPAR*α*, PGC-1a, CPT-1, UCP2, and UCP3) were significantly increased in ruscogenin-treated HFD-fed hamsters. Conversely, ruscogenin decreased the expression of genes involved in lipogenesis (SREBP-1c, ACC, and FAS). These results suggest that ruscogenin reduces hepatic lipid accumulation in two ways: downregulating lipogenic proteins and upregulating proteins in *β*-oxidation pathway.

## 5. Conclusion

We demonstrated that ruscogenin has a beneficial effect in inhibiting fat accumulation in liver, improves insulin resistance, inhibits inflammation, and possesses a repressive property on hepatic lipogenesis; these effects are associated with the inhibition of NF-*κ*B and SREBP-1c and induction of PPAR*α*. Therefore, ruscogenin could represent a promising agent to reduce fatty liver or reverse hepatic disorders linked to type 2 diabetes as a monotherapy or in combination with other existing drugs.

## Figures and Tables

**Table 1:** Effects of ruscogenin on body weight, liver and visceral fat weight, and feeding behaviors of hamsters.

	RCD-fed	HFD-fed			
	Vehicle	Vehicle	Ruscogenin (mg/kg/day)		
			0.3	1.0	3.0
Final body weight (BW) (g)	127.1 ± 9.1^d^	164.3 ± 7.1^b^	158.4 ± 8.1^b^	153.9 ± 6.3^b^	147.3 ± 7.3^a,c^
Epididymal WAT (g/100 g BW)	1.6 ± 0.3^d^	2.5 ± 0.3^b^	2.1 ± 0.4^b^	1.9 ± 0.2^d^	1.8 ± 0.3^d^
Perirenal WAT (g/100 g BW)	0.8 ± 0.2^c^	1.2 ± 0.2^a^	1.1 ± 0.3	1.1 ± 0.2	0.9 ± 0.2
Liver weight (g/100 g BW)	4.8 ± 0.3^c^	5.7 ± 0.3^a^	5.5 ± 0.3^a^	5.2 ± 0.3	5.1 ± 0.3
Food intake (g/day)	9.9 ± 1.3	11.0 ± 1.3	10.7 ± 1.8	10.9 ± 1.4	9.8 ± 1.5
Water intake (mL/day)	12.6 ± 4.4	13.2 ± 6.2	13.0 ± 4.5	12.6 ± 5.9	13.1 ± 4.3

The vehicle (distilled water) used to prepare the tested medication solution was given at the same volume. Values (mean ± SEM) were obtained from each group of 8 animals after 8 weeks of the experimental period. ^a^ *P* < 0.05 and ^b^ *P* < 0.01 compared to the values of vehicle-treated RCD-fed hamsters in each group, respectively. ^c^ *P* < 0.05 and ^d^ *P* < 0.01 compared to the values of vehicle-treated HFD-fed hamsters in each group, respectively.

**Table 2:** Effects of ruscogenin on insulin sensitivity, plasma lipid profile and free fatty acid, and hepatic lipids of hamsters.

	RCD-fed	HFD-fed			
	Vehicle	Vehicle	Ruscogenin (mg/kg/day)		
			0.3	1.0	3.0
Plasma glucose (mg/dL)	92.2 ± 3.8^d^	156.4 ± 4.1^b^	143.6 ± 4.1^b,c^	139.6 ± 3.9^b,c^	130.5 ± 2.9^b,c^
Plasma insulin (mU)	21.7 ± 0.3^d^	40.8 ± 0.5^b^	37.6 ± 0.4^b,c^	32.2 ± 0.3^b,c^	29.0 ± 0.3^a,d^
HOMA-IR	4.9 ± 0.2	15.7 ± 0.2^b^	13.3 ± 0.3^b,c^	11.1 ± 0.3^b,c^	9.5 ± 0.3^b,d^
Plasma TC (mg/dL)	115.1 ± 3.1^d^	257.8 ± 4.4^b^	196.2 ± 5.1^b,c^	178.0 ± 3.9^b,c^	155.8 ± 4.2^a,d^
Plasma TG (mg/dL)	90.2 ± 2.1^d^	157.0 ± 3.9^b^	130.9 ± 4.1^b,c^	118.6 ± 3.1^a,c^	109.7 ± 3.3^a,d^
Plasma LDL (mg/dL)	49.0 ± 3.8^d^	197.2 ± 5.4^b^	132.9 ± 6.9^b,c^	113.7 ± 6.1^b,c^	90.1 ± 5.4^a,d^
Plasma HDL (mg/dL)	48.1 ± 3.0^c^	29.9 ± 2.9^a^	37.1 ± 3.1^a,c^	40.5 ± 4.1^a,c^	43.7 ± 3.6^c^
Plasma FFAs (mg/dL)	29.9 ± 3.3^d^	60.2 ± 4.4^b^	53.7 ± 4.2^b^	48.0 ± 3.7^a,c^	37.2 ± 3.5^a,d^
Hepatic TC (*µ*mol/g liver)	9.9 ± 0.8^d^	19.2 ± 1.4^b^	17.3 ± 1.1^b^	15.5 ± 0.9^b,c^	13.7 ± 1.4^a,c^
Hepatic TG (*µ*mol/g liver)	8.6 ± 0.8^d^	17.0 ± 1.7^b^	15.7 ± 1.8^b^	12.3 ± 1.9^b,c^	11.0 ± 1.2^a,c^

The vehicle (distilled water) used to prepare the tested medication solution was given at the same volume. Values (mean ± SEM) were obtained from each group of 8 animals after 8 weeks of the experimental period. ^a^ *P* < 0.05 and ^b^ *P* < 0.01 compared to the values of vehicle-treated RCD-fed hamsters in each group, respectively. ^c^ *P* < 0.05 and ^d^ *P* < 0.01 compared to the values of vehicle-treated HFD-fed hamsters in each group, respectively.

**Table 3:** Effects of ruscogenin on scores for hepatic steatosis and necroinflammation of hamsters.

	RCD-fed	HFD-fed	
	Vehicle	Vehicle	Ruscogenin (3.0 mg/kg/day)
Steatosis (scores 0–4)	0 ± 0^d^	3.1 ± 0.6^b^	1.7 ± 0.3^b,d^
Necroinflammation (scores 0–3)	0 ± 0^d^	1.1 ± 0.3^b^	0.6 ± 0.2^b,c^

The vehicle (distilled water) used to prepare the tested medication solution was given at the same volume. Values (mean ± SEM) were obtained from each group of 8 animals after 8 weeks of the experimental period. ^a^ *P* < 0.05 and ^b^ *P* < 0.01 compared to the values of vehicle-treated RCD-fed hamsters in each group, respectively. ^c^ *P* < 0.05 and ^d^ *P* < 0.01 compared to the values of vehicle-treated HFD-fed hamsters in each group, respectively.
